# Reducing Disparities for Women and Minority Business in Public Contracting Work: A Call for Social Virtuousness

**DOI:** 10.3389/fpsyg.2019.01264

**Published:** 2019-06-04

**Authors:** Audrey J. Murrell, Ralph Bangs

**Affiliations:** ^1^ College of Business Administration, University of Pittsburgh, Pittsburgh, PA, United States; ^2^ University of Pittsburgh, University Center for International Studies, Pittsburgh, PA, United States

**Keywords:** gender, entrepreneurship, disparities, virtuousness, equality, entrepreneurship

## Abstract

Despite government devoting time and resources to ending discrimination, disparities based on gender, race, and disadvantaged business status persist in the area of business development, access to capital, and contracting opportunities. We join with the growing number of scholars that call for the concept of virtuousness to be highly placed on the business and management research agendas. This research utilizes critical participatory action research (CPAR) as a tool for building capacity to define and implement meaningful change that has the potential to correct these persistent disparities. We describe a longitudinal project that uses CPAR for addressing gender and racial disparities in local government contracting opportunities. We developed a collaboration with several community, women and minority-serving and legal partners in order to move beyond documenting the problems and toward advancing corrective social policy changes based on the key principles of the CPAR methodology. We described this work in the context of social virtuousness and discuss the implications for future research and public policy.

## Introduction

Despite government devoting time and resources to ending discrimination, disparities based on gender, race, and disadvantaged business status persist in the area of business development, access to capital and contracting opportunities (MBDA, 2016). These persistent barriers to success for women (WBEs) and minority (MBEs) owned businesses can represent a breach in the social contract that government should support the betterment of all members of society regardless of dimensions such as gender, race, social class, etc. (Delcampo et al., 2010). While some efforts to combat these persistent disparities have been attempted, the lack of transformative and sustainable change suggests that perhaps different approaches are necessary. We extend the concepts of organizational virtuousness (Cameron, 2003) and positive organizational scholarship (Cameron et al., 2003) to the notion of “social virtuousness” which argues for the persistent needs of organizations, systems, and governments to actively engage in policies and practices that erase persistent inequities based on factors such as gender, race, social class, and other protected demographic categories. This notion, social virtuousness, provides the context for utilizing different approaches to address the persistent discrimination faced by women and minority businesses.

We join with the growing number of scholars that call for the concept of virtuousness to be highly placed on the business and management research agendas (Morse, 1999; Schudt, 2000; Park and Peterson, 2003). This suggests that social virtuousness can best be achieved by moving beyond the mere documentation of disparities toward engaging key stakeholders in the transformative work of eliminating inequities. Our work uses critical participatory action research (CPAR) as a tool for building capacity to define and implement meaningful change that has the potential to correct these persistent disparities. We describe a longitudinal project that uses CPAR for addressing gender and racial disparities in local government contracting opportunities. We conducted our work in collaboration with several community, women and minority-serving and legal partners in order to move beyond documenting the problems and toward advancing corrective social policy changes based on the key principles of the CPAR methodology. We describe this work in the context of social virtuousness and discuss the implications for future research and public policy.

## The Need for Social Virtuousness for Women and Minority Owned Businesses

Our research views gender and racial disparities within the broader context of virtuousness because of its focus on the behaviors, systems, practices, and outcomes that support the pursuit of positive human and organizational conditions (Cameron, 2003). This notion points to the responsibility of individuals, organizations, and society to adhere to high qualities and characters that support social betterment, harmony, and excellence (Jordan and Meara, 1990; Baumeister and Exline, 1999). Actions taken by policymakers, leaders, and government officials should have as their aim to enhance inclusive excellence rather than maximize individual gain or instrumental reciprocity (Peterson and Seligman, 2004). The perspective shifts our focus away from issues related to competition and profitability and toward the need to remove barriers that interfere or prevent positive outcomes for any segment within society (Fowers and Tjelveit, 2003). This is consistent with extensive research on positive organizational scholarship which attempts to maximize social betterment for individuals and organizations (Cameron et al., 2004; Bright et al., 2006). Thus, actions, policies, and structure that systematically prevent positive outcomes such as social betterment and excellence could be viewed as a breach of the social and psychological contract concerning social virtuousness values (Delcampo et al., 2010). The notion of the social and psychological contract has been shown to capture how and why people respond in different ways when they perceive that organizations (and others) fail to meet obligations established in a social exchange relationship (Shen et al., 2019).

Access to contracting and procurement opportunities within the public sector represents a multibillion-dollar business for women and minority-owned businesses. Lack of access to these opportunities has dramatic implications for the overall health, well-being, and sustainability of these diverse enterprises. Previous research shows that gender and racial gaps in business enterprise negatively impact productivity and economic outcomes at both the individual and the societal levels (Cuberes and Teignier, 2016). They estimate that the expected income loss from excluding 5% of women employees is about 2.5% but that percentage doubles if these women are owners/employers. These “entrepreneurial gaps” result in substantial economic losses for people and nations and varies by level of economic development across regions. This discrimination and persistent disparities in access to opportunities negatively impact social virtuousness for these diverse business owners.

While some gains have been achieved, disparities persist, and traditional methodological approaches are often limited (Bangs et al., 2007). For example, legal standards often call for the use of disparities studies in order to provide evidence of discrimination before any preventative measures or change efforts are implemented. Some argue that this legal requirement has created a new industry of consulting firms devoted to conducting disparity studies yet most of these studies do little to outline strategies for change (Celec et al., 2002). In addition, disparity studies are merely descriptive in nature and often ignore the real experiences and perspectives of women and minority business owners. These limitations in traditional research approach can act as barriers to identify meaningful strategies for positive change. For example, after spending a half million dollars on a disparity study and receiving nine volumes of reports, the City of Pittsburgh’s elected officials ultimately rejected the study’s findings and no action was taken. Thus, using methods that have social virtuousness as its underlying principles is important for creating positive social change for these historically disadvantaged businesses.

## Beyond Disparity Studies for Reducing Inequities for Women and Minority Businesses

The U.S. Minority Business Development Agency (MBDA), in a review of 100 disparity studies, found that the median share of local and state government contract dollars awarded to minority owned business enterprises (MBEs) was just 19% of their overall shares of available businesses in the relevant product and geographic markets (MBDA, 2016)^1^. More recent disparity studies for local governments attempt to provide evidence for underutilization of women and minority owned business enterprise shares of dollars awarded relative to their national or local representation. For example, women and minority entrepreneurs were substantially underutilized for City of Charlotte contracts (Charlotte Management and Financial Services, 2018). Another study found that women and minority business enterprises were substantially underutilized as subcontractors in Atlanta Housing Authority contracts (Keen Independent Research, 2017a). This finding was replicated in another disparity study that found that women and minority businesses received just 60% of contract dollars awarded by Hennepin County (Minneapolis) compared to their availability for contracts (Keen Independent Research, 2017b). Consistently, these disparity studies find statistically significant and substantial underutilization of W/MBEs occurred in all procurement categories (Griffin and Strong, 2018).

While disparities in overall utilization are often identified, key issues such as a firm’s willingness, availability, and qualifications for doing public contracting work are often overlooked or ignored (Bangs et al., 2004). In addition, these disparity studies are conducted by external consultants who have no investment or motivation to solve the systemic issues that lead to these persistent inequities. As a result, the problem continues and social betterment remains elusive.

This suggests that a different methodological approach may be needed in order to better understand, document, and outline strategies for the prevention of discrimination against women and minority business enterprises.

Another important aspect of this issue is the cost to individuals and to society for persistent disparities and discrimination against women and minority businesses. Previous work has developed models for documenting the negative economic impact of persistent gender and racial inequalities (Mitra et al., 2014). This work documents the negative impact of persistent discrimination as well as the positive impact on economic development when these persistent disparities are reduced.

Thus, the extensive number of local and national disparity studies provides convincing evidence that bias and discriminatory practices negatively impact access to opportunities for women and minority own businesses. These macro-level analyses are also paired with economic analyses that show the negative impact on financial capital of these entrepreneurial gaps. While valuable, there are several limitations with relying on disparity studies alone to address these gaps for women and minority businesses. First, it is unclear from prior disparity studies to what extent lack of bidding was a reason why W/MBEs were not obtaining government contracting work. This is an important limitation that should be addressed in order to provide clear and compelling evidence of systematic discrimination. Including this factor also addresses the counter argument that there are not enough qualified and available women and minority firms for contracting work. Second, while disparity studies show differences in the outcomes, some argue that women and minority entrepreneurs often do not submit bids for large-scale contracting work because of the time, costs, and lack of confidence in the contract bidding process (Bangs et al., 2007). Third, the need to identify what legal scholars refer to as “sympathetic plaintiffs” is necessary to not simply document discrimination against women and minority firms, but to also take some type of corrective or punitive action (Bangs and Murrell, in press). This suggests that a different methodological approach is necessary to move beyond simply documenting that disparities exist in outcomes for W/MBEs but make an actionable case that is followed by some type of meaningful policy change toward the goal of social betterment.

## Critical Participatory Action Research as a Tool for Social Virtuousness

The practice of critical participatory action research (CPAR) focuses on the use of university research in collaboration with community-based organizations in order to impact and change social policy (Sandwick et al., 2018). Our work provides a specific illustration of CPAR that aims to influence social policy surrounding the utilization and inclusion of W/MBEs in local government public contracting. Our application of CPAR within the current context is warranted given the need to move beyond disparity studies, and this approach has a long history of examining social issues such as work on racial differences in urban policing (Stoudt et al., in press), severity of disciplinary actions based on race, gender, and sexuality (Chmielewski et al., 2016), inequity in education access and feelings of psychological safety based on sexual orientation (Birkett et al., 2014), and minority youth activism (Stoudt et al., 2016).

Scholars in this field argue that the use of CPAR as a research methodology in areas related to diversity and inclusion is critical in order to balance inequities in power, privilege, and hierarchy as well as academic biases that can impact the formation, design, and interpretation of academic research (Torre, 2009). A core tenant of CPAR relevant to examining minority contractors is giving traditionally marginalized communities the right to both participate in and shape research and policy formation that impacts their lives (Appadurai, 2006). This inclusion of traditionally excluded communities is consistent with the values of social virtuousness and positive organizational scholarship (Cameron, 2003). Thus, we argue that research seeking to inform, impact, and change social policy must attend to the necessity for inclusion of those diverse voices who are most impacted by the outcomes of this research.

Our work focuses on reducing barriers for W/MBEs as it relates to contracting opportunities with public or governmental organizations. This research seeks to not only document the issues of discrimination that produces inequities in access and utilization but also to put in place policies and processes that can help reduce these negative conditions. The use of CPAR as a methodological framework is a good fit for this context because it engages those that are most affected by persistent discrimination and also builds capacity for social change by engaging community partners as co-creators of meaningful policy change (Massey and Barreras, 2013). It also means that our conclusions and recommendations are structured in a way that shapes evidence-based policy making by utilizing inclusive research methodologies for producing transformative social change (Nelson, 2013).

**Figure 1:**
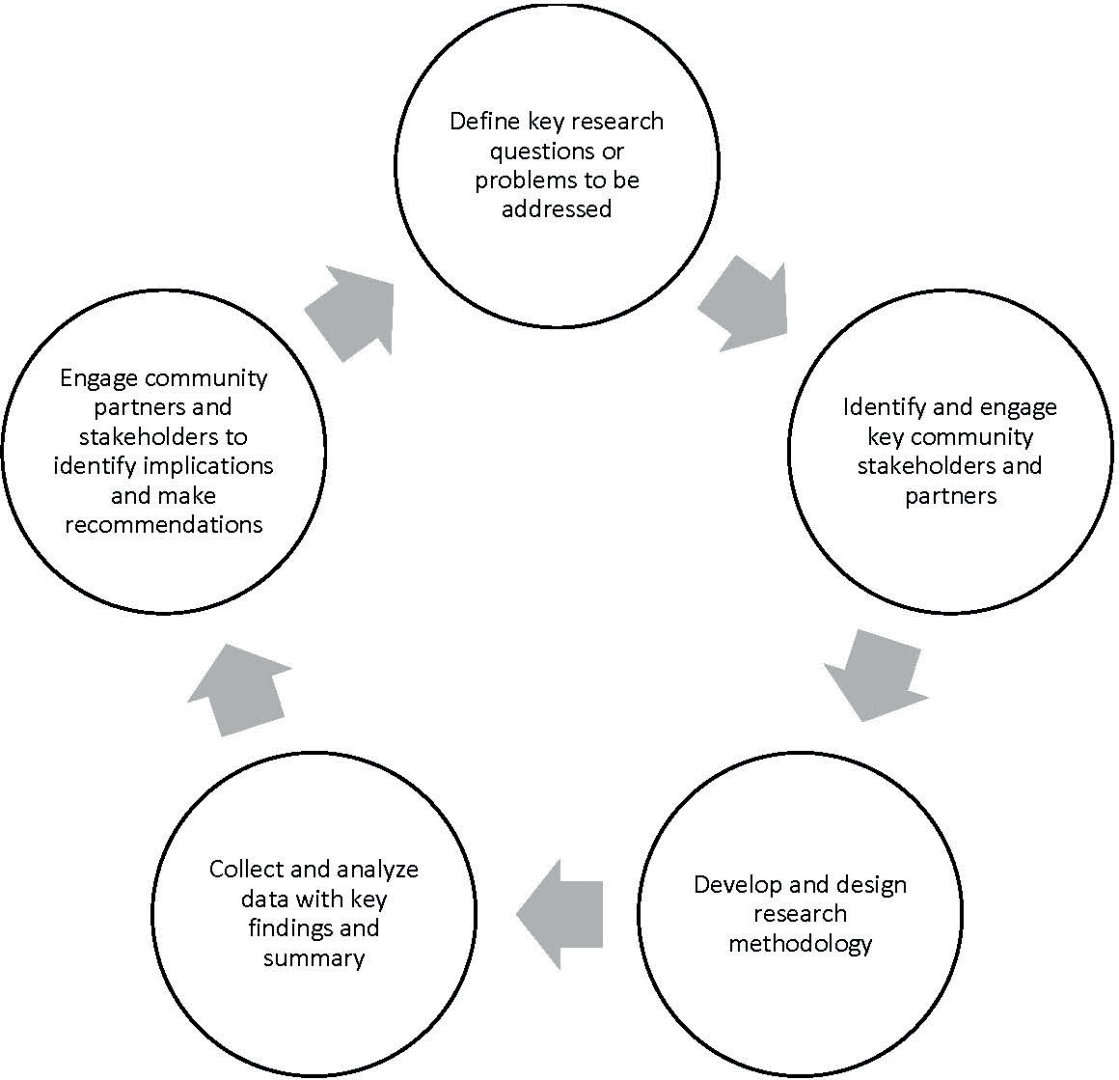
Overview of the critical participatory action research approach.

We summarize a series of participatory action research projects that examine potential explanatory factors for persistent disparities utilizing a community-academic collaborative effort to bring about meaningful policy change. The long-term goal is to provide the structure, process, and outcomes that are in line with the notion of social betterment and virtuousness for women and minority business owners. Our insights and recommendations on how to utilize participation action research to impact social policy in the area of contracting discrimination are discussed along with areas for future research. Figure 1 provides an overview of the CPAR process across five key stages: (1) define research question, issues and/or problems to be addressed; (2) engage community partners to provide input on question and approach; (3) design research methodology and data collection approach; (4) analyze data and summarize key findings; (5) collaborate with community partners on interpretation and application of findings. These phases can be repeated as needed in order to address the complexity of different issues and/or research questions.

## Using Critical Participatory Action Research

In Phase 1, we decided to study this issue in relation to W/MBEs in the local Pittsburgh area with a specific focus on prime contracts awarded by the two main government entities, the City of Pittsburgh and Allegheny County. We shaped our initial action research around three key questions based on our discussions with community-based partners:

1. To what extent are certified W/MBEs qualified for local government prime contracts of $25,000 or more?
2. To what extent do certified W/MBEs submit bids for these prime contracts?
3. Why do not certified W/MBEs submit bids for these prime contracts?

Our methodology was structured to help answer these three key questions in a manner that not only provided valid evidence of discrimination, but also collected meaningful first-person experiences from women and minority business owners who are both qualified and willing to engage in public contracting work. We also engaged key community, non-profit, and legal experts in the shaping of our approach that includes input on survey questions, recruitment of participants, and review of initial outcomes. With input from these partners, we identified a specific set of W/MBE firms that had completed contracts of $25,000 in the last 3 years were engaged within the procurement category for at least one of the local government prime contract opportunities and/or has submitted bids during the review period. This methodology provides strong rigor for qualitative research in the areas of credibility and confirmability. Given that our framing of the questions and review of findings were discussed with community stakeholders and participants, we are able to confirm that our findings are believable (credible) and corroborated (confirmability) using the CPAR methodology.

Thus, in our initial study, we:

1. Identified 131 prime contract opportunities of $25,000 or more at the City of Pittsburgh, Allegheny County, and their authorities
2. Identified 91 certified MBEs in the county in the same industry as one or more of the 131 contract opportunities
3. Made 384 matches between 65 contract opportunities and 91 local MBEs
4. Provided information on the contract opportunities to the matched MBEs
5. Tracked 108 bid openings with at least one bid submitted, 376 bids submitted, and 101 prime contracts awarded
6. Surveyed certified MBEs and 102 (55%) out of 184 reported reasons for not bidding

Our initial findings were that 123 MBEs had completed contracts of $25,000 in the last 3 years, and 91 were in the same procurement category as at least one of the local government prime contract opportunities. In addition, MBEs submitted 3.2% (12) of the 376 bids, submitted 3 of the 101 low bids, received 3% of the contracts (3 of 101), and received 2.3% of the dollars awarded.

In addition to capturing data on overall submits and participation levels, we also were advised to collect survey data to provide more details on what experiences and factors were seen by the minority contractors as the most significant barriers that they faced. Of the 72 minority business orders completing our survey, they gave these reasons for not submitting bids:

1. 55% – do not have the right contacts (contracts “wired” for some firms; get early info.)
2. 49% – difficult getting information (e.g., bid information is sent too late or not at all)
3. 46% – difficult to get bonding
4. 44% – too expensive to prepare bids
5. 38% – unsuccessful in the past
6. 31% – perceive the process to be unfair
7. 17% – lack technical knowledge to submit bids
8. 16% – were not paid from previous contracts

Other reasons for not bidding were: lack of working capital, slow pay, lack of time and resources, supplier prices change during contract and lose money on contract, and white firms were awarded more than their low bids. Based on discussions with our research partners, we concluded that:

1. Lack of MBE bidding is a major reason for lack of MBE prime contracts with local government
2. No support for the notion that there are no “qualified” MBE firms for prime contracts
3. Perceived and actual access to bidding information and key social networks are important
4. Determining a pattern of discrimination in prime contract decisions by local government will require increasing bidding by qualified MBEs so there are sufficient cases to study.

In addition to capturing data on overall submits and participation levels, we also were advised by our partners to collect survey data to provide more details on what experiences and factors were seen by the women and minority contractors as the most significant barriers that they faced. Frequent reasons for not submitting bids including lack of access to information, barriers to securing necessary bonding, cost/expense of preparing bids, lack of previous success, and perceived unfairness of the process. Other less frequent reasons for not bidding were: lack of working capital, slow payments from local government, lack of time and resources, supplier prices change during contract and lose money on contract, and white firms were awarded more than their low bids. We engaged our community and research partners in a dialog about these findings along with ideas and strategies for helping to reduce these barriers for W/MBEs receiving contracts.

Based on these conversations with community partners, we outlined several policy implications of this study for changes to local government policies and procedures. These included how contracts were structured, publicly advertised, monitored, and awarded. In addition, these recommendations identified the need to implement adequate tracking systems by local government to insure accountability of payments and participation once contracts were awarded. More long-term solutions identified included expanding networking opportunities, mentoring, and capacity building efforts for W/MBEs.

We shared our findings, methodology and recommendations in several of meetings with our community partners. Our report was also disseminated publicly to stimulate further dialog as well as to honor our commitment to the research participants that our findings would be widely shared. Based on their feedback and input from our interactions with the MBEs, the need to look not only at contracts awards but also change orders was identified. Thus, we undertook a second participatory research study to look more deeply into change orders in one public contracting agency within local government. Our use of CPAR and the outcomes of Phase 1 identified this as a necessary next step.

## Prime Contracts and Change Orders Awarded to Women/Minority Owned Business Enterprises

In the first 9 months of Phase 2, we gathered data on prime contracts of $10,000 or more awarded by one local government, the local school district (Pittsburgh Public School or PPS). The purpose was to determine MBE shares of contracts and contract dollars but to also look at changes to contract terms and amounts (change orders) once the official award was made. We collected this information by examining official organizational minutes, including reports, resolutions, and updates *via* the public website. We organized the data according to department (e.g., facilities and general services) and whether the approvals were for original contracts or for change orders. Once again, we identified firms certified as W/MBEs among the approved firms that insuring they were qualified for the contracting work. The resulting list of W/MBEs with board-approved prime contracts was then cross-checked against the list of PPS contracts awarded as provided by the PPS Minority/Women Business Department as key government officials.

Including an examination of change orders as identified by our community-based partners yielded important differences in the results of overall W/MBE under-utilization. This led to additional recommendations to track not only contract awards but also actual pend and overuse of change orders which disproportionately impact W/MBEs. We also interviewed contracting officials of local government along with 20 certified MBEs that the PPS Women/Minority Coordinator would like to get to know better or have them submit more bids.

We asked the 20 certified MBEs during a 1-h (notes were taken and then transcribed) engagement interviews a set of standard questions:

1. Could you tell us about the strengths of your company and your vision for the company?
2. What work have you done in recent years with PPS? In what ways has the experience been positive or negative?
3. Are you interested in obtaining prime contracts with PPS? Why or why not?
4. In what ways have you tried to find out about prime contract opportunities with PPS? What were the results of these efforts?
5. What would help you to increase your bidding for prime contracts with PPS?

Examining the content of these interviews by both authors (until consensus on key and/or reoccurring themes were identified) revealed that most of the W/MBEs had many contracts in the public and private sectors but were doing business outside of the immediate local area because of lack of access to opportunity and past experiences with barriers to contract opportunities. Of the 20 W/MBEs interviewed, a third had received prime contracts from PPS, but the rest had either tried but not received prime contracts from PPS or not tried at all. The finding that two-third of the firms that were qualified and interested in prime contract opportunities were not able to be selected provides strong evidence of under-utilization of W/MBE contractors who were qualified for prime level opportunities. In fact, a majority (90%) of the W/MBEs indicated a desire to have greater prime contracts or large-scale opportunities with local government. This provides evidence that there were qualified W/MBEs who were available and interested in more work with local government, especially involving prime contracts. This supports the feedback we received from our meetings with community partners in which the idea of lack of availability of qualified, available and interested W/MBEs was frequently not a legitimate reason for the lack of diverse business participation in contracting work.

Thus, we found that the W/MBEs were proactively looking for business and used a variety of methods to try to find out about prime contract opportunities. Despite these proactive efforts, W/MBEs reported that they had difficulty obtaining prime contract opportunities. When we discussed these obstacles, W/MBEs reported a range of issues that could be addressed by changes in policy and process for local government. In addition, lack of access to information and contacts within local government (the notion of closed networks) were frequently mentioned as barriers for these business owners.

Examining the content of these interviews revealed that most of the MBEs had many contracts in the public and private sectors but were doing business outside of the local area because of lack of access to opportunity. Of the MBEs interviewed, eight had received prime contracts from PPS, but seven had tried but not received prime contracts from PPS, and five had not tried. Thus, there was evidence of under-utilization of minority contractors who were qualified by PPS. Lastly, a majority of the MBEs (19 of the 20) indicated a desire to have prime contracts or more prime contracts with PPS. This provided evidence that there were qualified MBEs who were available and interested in more work with PPS, especially involving prime contracts. This contradicts the feedback we received from PPS during our meetings with community partners in which the idea of lack of availability of qualified, available and interested MBEs was frequently cited as the reason for the lack of minority participation in contracting work.

As part of the CPAR methodology, we then asked our selected W/MBE participants to provide their input and insight into areas for improvement to both policy and business process. To submit more bids, W/MBEs indicated that PPS staff need to be less opposed to supplier diversity and that bid and proposal specifications need to be more accurate and transparent (i.e., access to information). These business owners also suggested that large contracts should be broken into smaller parts to increase the likelihood that they would be able to submit bids. In addition, they suggested that excessive change orders need to be curtailed especially for work involving small contractors and W/MBEs.

Based on our work with community partners, collecting of data and inclusive feedback from our W/MBEs, we then constructed a set of recommendations that would be used to shape policy and process for local government. We shared these findings with our local government, W/MBE participants and made them accessible in a series of reports that were available to the public.

## Impact of Critical Participatory Action Research on Women/Minority Owned Business Enterprises

Our research findings together with advocacy by our legal and political partners in part resulted in the adoption of a number of changes in policies by local government which are still in place years after the completion of Phase 2. The leadership within local government directed the creation of a strategic plan supported by administrative rules and procedures to ensure that W/MBEs had greater access to and equal opportunity to participate contracting work. This plan contained a clear mission statement toward diversity and inclusion, along with specific goals, strategies, and performance measures. Thus, one local government was impacted by our CPAR work by developing what they labeled their Business Opportunity Plan^2^. This plan included several administrative rules and procedures what were required to be addressed. The changes included two main categories: supporting W/MBE capacity building and strengthening the contracting process. Several specific recommendations were included (but not limited to) in each of these categories:

### Supporting Firm Capacity-Building

1. Providing or referring W/MBEs to appropriate resources as needed for technical and financial assistance
2. Conducting outreach to encourage new W/MBEs to bid on public contracting
3. Advertising in newspapers to ensure appropriate reach and frequency of potential W/MBEs per specific public contracting opportunities
4. Encouraging mentoring and joint ventures with other majority or minority firms
5. Providing a dedicated resource to manage the promotion, development, and growth of W/MBEs for the local government public contracting opportunities

### Accessibility in the Contracting Processes

1. Determining which government certifications reliably identify minority, women and socially and economically disadvantaged business ownership and control so participation can be accurately counted
2. Designing bid packages in such a way to promote rather than discourage participation
3. Accelerating contract awards as well as payments to prime and subcontractors
4. Including language in bid solicitations that clearly sets forth the objective of the policy and includes local government’s anti-discrimination clause
5. Providing quarterly reports of W/MBE participation that can be readily accessed on the website and communication materials
6. Maintaining a searchable W/MBE database that can be readily accessed on the website
7. Establishing an advisory committee to provide feedback and support of local government’s efforts
8. Ensuring that the job descriptions of leadership and managers within local government include a responsibility for understanding and adhering to the new W/MBE policy

## Discussion and Lessons Learned

Our research examines gender and racial disparities in access to contracting opportunities within the broader context of social as part of the broader notion of positive human and organizational conditions (Cameron, 2003). We join others by asserting that actions taken by policymakers, leaders, and government officials must enhance inclusive excellence rather than maximize individual gain (Peterson and Seligman, 2004) in order to maximize social betterment for individuals and organizations (Cameron et al., 2004; Bright et al., 2006).

Clearly, access to contracting and procurement opportunities within the public sector fits into this notion of virtuousness as part of overall social betterment. Contracting opportunities represents a multibillion-dollar business for women and minority owned businesses and lack of access threatens the economic sustainability of these diverse enterprises.

While some gains have been achieved, disparities persist and traditional methodological approaches are often limited (Bangs et al., 2007) in providing compelling evidence for systemic biases and exclusion. These traditional methodologies also do not engage key stakeholders who are not only effective by this social exclusion but could also serve as key advocates for social and organization change. Our work provides a number in insights on how to engage community-based partners in producing meaningful change in social and organizational policy concerning diversity and inclusion. Conducting interdisciplinary research means that the researchers are from different disciplines. To be successful, the researchers need to respect each other’s capabilities and ideas and have a good working relationship. This can come from knowing and valuing each other’s past work and spending time working together. Conducting diversity research means focusing on race, ethnicity, gender, LGBT, and/or other historically disadvantaged populations. This requires that researchers have expertise on diversity and use an evidence-based approved for shaping policy change within local government.

Our work has clearly had impact on local government policies in the case of Pittsburgh Public Schools because the research produced definitive evidence of racial discrimination and our legal and community partners were able to use the research to pressure PPS to change policies. However, policy change does not mean all of our recommended policies were adopted or implemented which is one of the limitations of this work. While we were able to engage community partners in the design, execution, and evaluation of this research, the ability of CPAR to change systemic biases is someone limited. A further limitation is that any recommendations for changes in policies may not be effective in reducing a diversity problem even if adopted and implemented since the researchers and community members are not part of the actual implementation process. While our use of CPAR provides a strong case for credibility as a dimension of rigor for qualitative research, issues of transferability are another limitation of the current work. While the inclusion and feedback from our participants provides validation that our results have strong credibility, our sample is specific to one city and thus may not be transferable to other contexts or locations. Being able to transfer our findings to a larger city or one with different economic or industry conditions is limited. Thus, a key lesson is that doing a one-off study is never enough. Researchers need to make sure that new and appropriate policies were adopted and implemented and that the policies were effective in improving conditions. The use of CPAR is a powerful approach to not only document where disparities and discrimination exist but also engage a set of stakeholders in the active process of policy and process change.

Our approach also helps us to move beyond the singular use of disparity studies. Traditional disparity studies can provide all of the information that local governments need to set goals for W/MBE participation in contracts, determine why goals are not being met, and identify policy changes for achieving these goals. However, these studies are expensive and should be done only if the political will exists to enact recommended policies. Further, disparity studies need to be repeated every 3–5 years to find out if policy changes were made and if the policies were effective.

If disparity studies are not done, then local researchers can conduct relatively low-cost studies that gather some important information and have an impact. These studies need to be ongoing to determine whether new policies were adopted and implemented, whether diverse owners of businesses increased participation in contracting, and what additional policy changes are needed. Our efforts are consistent with previous scholars in this field who argue that the use of CPAR as a research methodology in the areas of diversity and inclusion is critical in order to address issues of power, privilege, hierarchy and academic biases that can impact the formation, content, and interpretation of academic research (Torre, 2009). Using CPAR within this specific area of increasing utilization of MBEs in contracting work has provided additional support for this approach within diversity research. Thus, we provide the current research as another illustration of how this powerful technique can be used to enhance diversity research. For our work, CPAR is an important tool for informing as well as shaping social policy in a manner that is inclusive of those diverse voices most impacted by the outcomes of impactful research that creates lasting benefit for women and minority businesses.

## Ethics Statement

This study was carried out in accordance with the recommendations of University of Pittsburgh Institutional Review Board with written informed consent from all subjects. All subjects gave written informed consent in accordance with the Declaration of Helsinki. The protocol was approved by University of Pittsburgh, Institutional Review Board.

## Author Contributions

All authors listed have made a substantial, direct and intellectual contribution to the work, and approved it for publication.

### Conflict of Interest Statement

The authors declare that the research was conducted in the absence of any commercial or financial relationships that could be construed as a potential conflict of interest.

## Footnotes

^1^The MBDA reported only on MBEs, although disparity studies usually cover MBEs and WBEs. The MBDA did not report separately on local government although 59 of the 100 disparity studies were on local governments (41 were on states). About 96 of the 100 studies were released from 2004 to 2016; the other four were from 1994 to 2001.

^2^To date, this plan has not been released to the public.
